# Improving oxygenation in a patient with respiratory failure due to morbid obesity by applying airway pressure release ventilation: a case report

**DOI:** 10.1186/s13256-024-04665-2

**Published:** 2024-08-05

**Authors:** Ryosuke Nobe, Kenichiro Ishida, Yuki Togami, Masahiro Ojima, Taku Sogabe, Mitsuo Ohnishi

**Affiliations:** Department of Acute Medicine and Critical Care Medical Center, Osaka National Hospital, National Hospital Organization, 2-1-14, Hoenzaka, Chuo-Ku, Osaka, 540-0006 Japan

**Keywords:** Airway pressure release ventilation, Critical care, Mechanical ventilation, Morbid obesity, Respiratory failure

## Abstract

**Introduction:**

Morbidly obese patients occasionally have respiratory problems owing to hypoventilation. Airway pressure release ventilation is one of the ventilation settings often used for respiratory management of acute respiratory distress syndrome. However, previous reports indicating that airway pressure release ventilation may become a therapeutic measure as ventilator management in morbid obesity with respiratory failure is limited. We report a case of markedly improved oxygenation in a morbidly obese patient after airway pressure release ventilation application.

**Case report:**

A 50s-year-old Asian man (body mass index 41 kg/m^2^) presented with breathing difficulties. The patient had respiratory failure with a PaO_2_/F_I_O_2_ ratio of approximately 100 and severe atelectasis in the left lung, and ventilator management was initiated. Although the patient was managed on a conventional ventilate mode, oxygenation did not improve. On day 11, we changed the ventilation setting to airway pressure release ventilation, which showed marked improvement in oxygenation with a PaO_2_/F_I_O_2_ ratio of approximately 300. We could reduce sedative medication and apply respiratory rehabilitation. The patient was weaned from the ventilator on day 29 and transferred to another hospital for further rehabilitation on day 31.

**Conclusion:**

Airway pressure release ventilation ventilator management in morbidly obese patients may contribute to improving oxygenation and become one of the direct therapeutic measures in the early stage of critical care.

## Introduction

Morbidly obese patients are often prone to respiratory compromise owing to the weight of the anterior chest wall and compression on the diaphragm from increased intra-abdominal pressure [1, 2]. Airway pressure release ventilation (APRV) is one of the mechanical ventilation settings that can improve oxygenation by recruiting collapsed lung [3, 4], which is occasionally used for respiratory management of acute respiratory distress syndrome (ARDS) [4–7]. However, universally accepted indications of APRV, especially for cases of respiratory failure owing to morbid obesity, are unclear. Previous reports are limited in suggesting APRV may have become a direct treatment for hypoventilation syndrome caused by morbid obesity [8–10].

We report a case of respiratory failure owing to morbid obesity, presenting with severe atelectasis, in which oxygenation markedly improved after using APRV.

This paper does not require the consent of an ethics committee. In addition, the patient has been anonymized in accordance with the Personal Information Protection Law, and consent for publication has been obtained from the patient.

## Case presentation

The patient was a 50s-year-old Asian man with a height of 171 cm, weight of 120 kg, and body mass index (BMI) of 41 kg/m^2^, presenting with left diaphragmatic nerve palsy owing to trauma at the age of 18 years. Other medical history included sleep apnea syndrome, type 2 diabetes mellitus and hypertension. The patient spent much time in the dorsal position because of sleep apnea syndrome. One day, after going to work, the patient had difficulty breathing and was transported to our hospital.

**Fig. 1:**
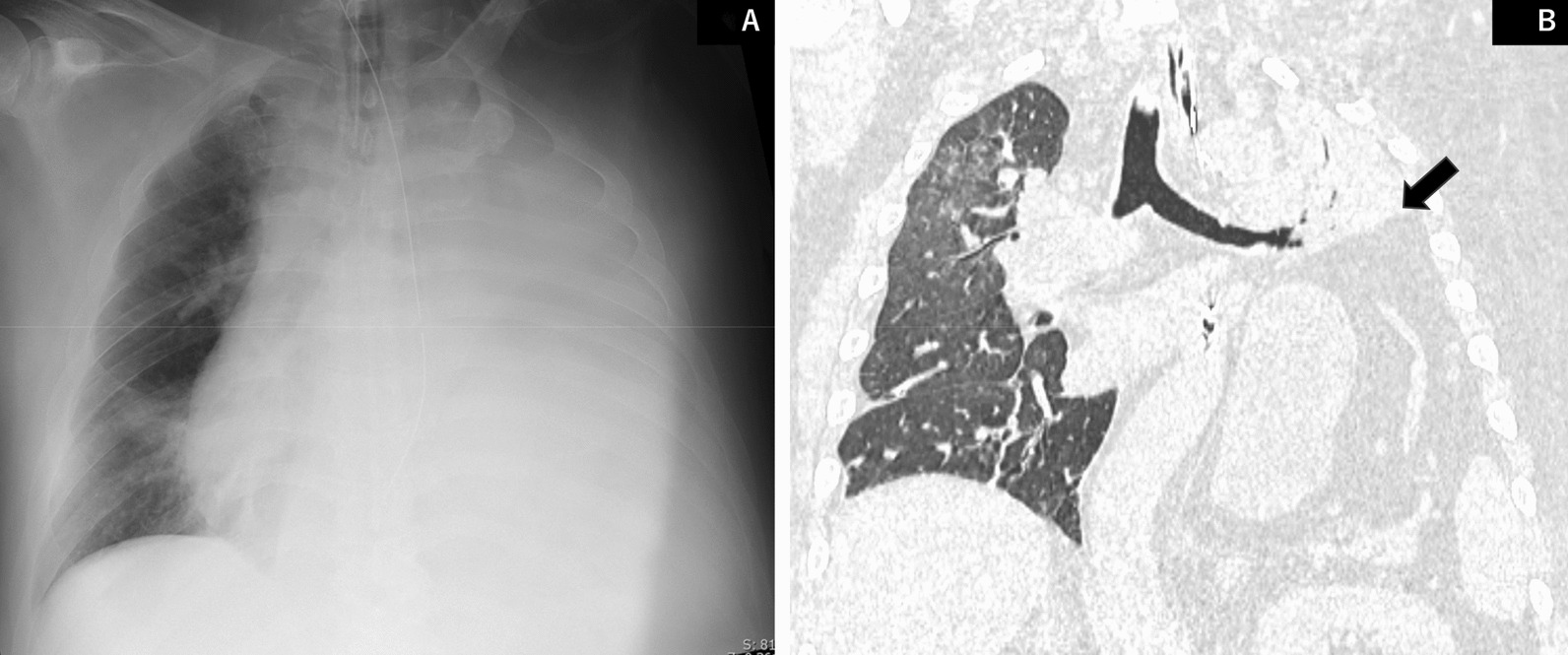
**A** Chest X-ray findings on admission showed loss of air content in the left lung. **B** Coronal computed tomography findings on admission showed slight ground-glass areas in the upper lobe of the right lung. The left lung showed almost complete loss of air content and elevated left diaphragm, which is indicated by a black arrow

On hospital arrival, the patient’s vital signs were as follows: Glasgow coma scale of E4V5M6, SpO_2_ of 87% (room air), respiratory rate pf 28 breaths/minute, blood pressure of 182/101 mmHg, pulse of 108/minute, and body temperature of 35.8 °C. As breathing was slightly effortful, left breathing sounds were diminished. Arterial blood gas analysis (oxygen 2 L/minute) showed a pH of 7.235, PaCO_2_ of 87.5 mmHg, PaO_2_ of 62.9 mmHg, and the blood test showed white blood cell (WBC) count of 7700/μL, C-reactive protein (CRP) 1.44 mg/dL, aspartate transferase (AST) 15 U/L, alanine transaminase (ALT) 15 U/L, Cr 0.69 mg/dL, Na 144 mEq/L, K 3.6 mEq/L, Hb 16.7 g/dL, albumin (Alb) 4.2 g/dL. Chest X-ray revealed loss of air in the left lung (Fig. 1A). Plain computed tomography showed slight ground-glass areas in the upper lobe of the right lung, besides the left lung showed almost no air content, and the left diaphragm was elevated (Fig. 1B).

**Fig. 2:**
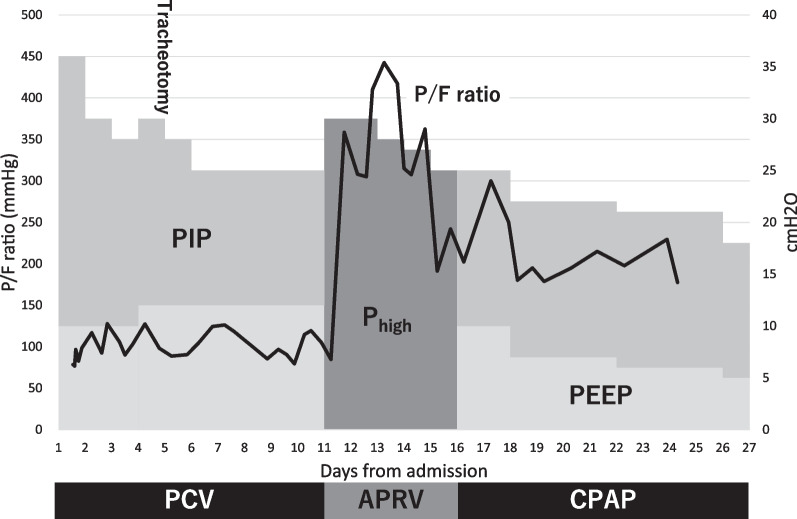
PaO_2_/F_I_O_2_ ratio and progress of ventilator settings after admission. The black line expresses PaO_2_/F_I_O_2_ ratio, and dark and light gray areas indicate peak inspiratory and positive end-expiratory pressure, respectively PIP, peak inspiratory pressure; P_high_, high pressure; PEEP, positive end-expiratory pressure; PCV, pressure control ventilation; APRV, airway pressure release ventilation; CPAP, continuous positive airway pressure

The patient’s respiratory condition worsened after arrival; he was diagnosed with type 2 respiratory failure owing to atelectasis and pneumonia related to morbid obesity, and was intubated and managed on a ventilator (Evita V 300; Dräger Medical AG & Co.) in the intensive care unit. The ventilator was set to pressure control ventilation (PCV) with F_I_O_2_ of 0.8, peak inspiratory pressure of 36 cmH_2_O, inspiratory time of 0.95 seconds, positive end-expiratory pressure (PEEP) of 8 cmH_2_O, and ventilation rate of 22/minute. Although a high drive pressure of 28 cmH_2_O was initially required to maintain adequate minute volume, hypercarbia improved relatively quickly. Subsequently, inspiratory pressure was adjusted to around 30 cmH_2_O to maintain a tidal volume of 0.6–0.8 mL/kg (ideal body weight). However, hypoxia persisted with a PaO_2_/F_I_O_2_ ratio (P/F ratio) of approximately 100 (Fig. 2).

**Fig. 3:**
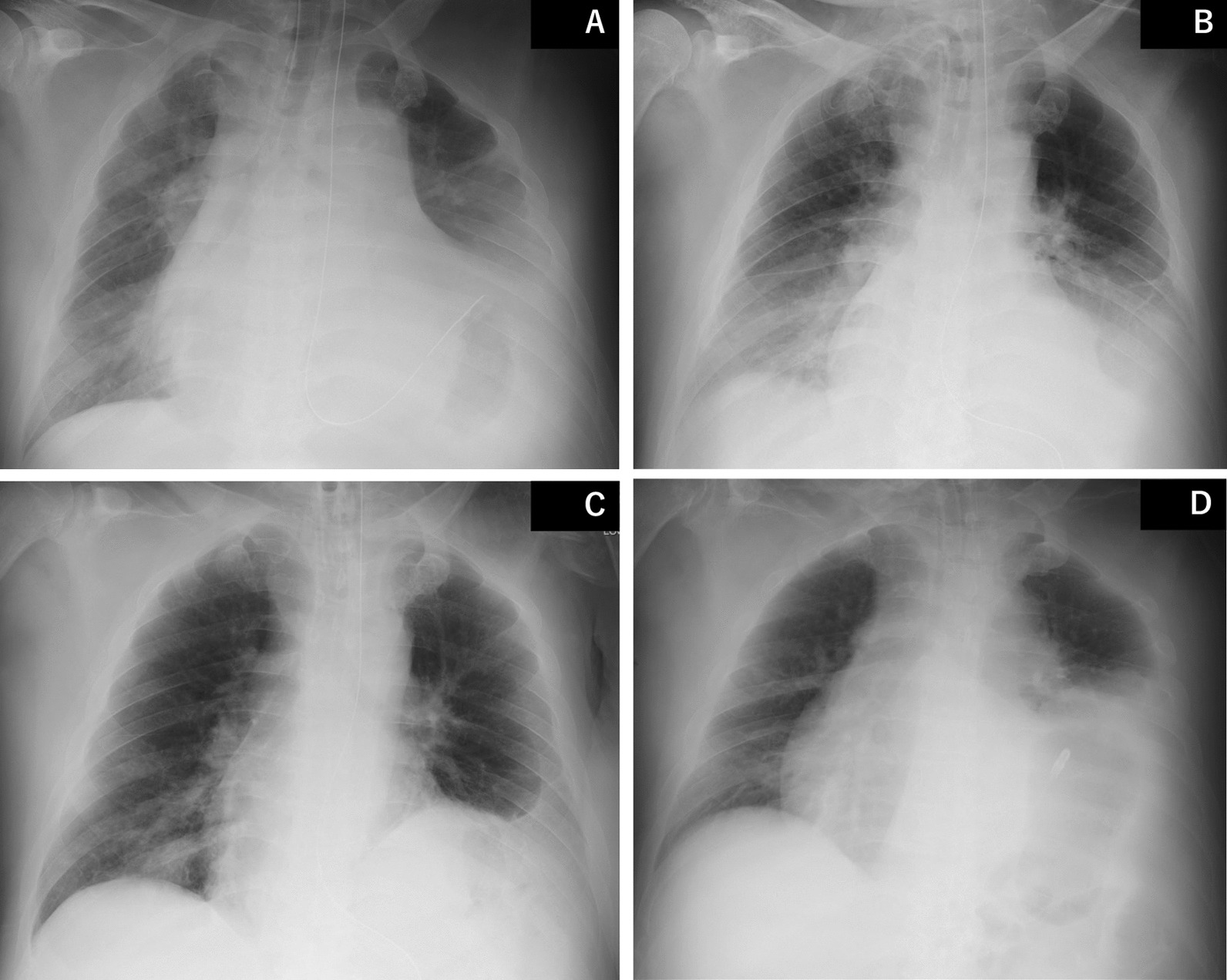
**A** Chest X-ray on day 2 after bronchoscopy; **B** Chest X-ray on day 7; **C** Chest X-ray on day 11; **D** Chest X-ray on day 29 before ventilator withdrawal

On day 2, bronchoscopy was performed to atelectasis, and chest X-ray showed that air content in the left lung improved slightly (Fig. 3A); however, oxygenation did not improve. Tracheotomy was performed for further positional changes and respiratory rehabilitation on day 5. On day 7, chest X-ray showed that the left diaphragm had descended, and permeability of the left lung had improved. However, the right lower lobe had an enhanced shadow (Fig. 3B), and oxygenation did not improve. On day 11, the ventilator was shifted to APRV mode (P_high_/P_low_ = 30/0 cmH_2_O, T_high_/T_low_ = 6/0.5 seconds) with the expectation of alveolar recruitment effect. This markedly improved hypoxia, with a P/F ratio of 300 approximately 3 hours after changing the setting while maintaining the tidal volume. Chest X-ray performed on day 12 revealed improved lung permeability (Fig. 3C). After changing the ventilator setting to APRV, the patient underwent active rehabilitation under light sedation. On day 16, the ventilator setting was switched to continuous positive airway pressure mode, and the patient remained well-oxygenated and showed no carbon dioxide retention. The patient was weaned from ventilator on day 29, and the X-ray finding showed that although the diaphragm was elevated, the patient’s respiratory status did not deteriorate (Fig. 3D). The patient was transferred to another hospital for further rehabilitation on day 31. Since admission, the patient’s weight has been controlled through rehabilitation and nutritional management, and his weight had decreased to 104 kg (BMI 36 kg/m^2^) at discharge.

## Discussion

The patient had an elevated diaphragm owing to morbid obesity and left diaphragmatic nerve palsy, requiring ventilator management owing to respiratory failure; however, oxygenation improved after applying APRV. We suggest that APRV has the potential to be an effective ventilator setting for respiratory failure owing to morbid obesity.

APRV is expected to improve oxygenation by increasing ventilation of aeration-dependent lung tissue, opening nonaerated tissue, and reducing dead space in the lungs; consequently, this would improve the alveolar recruitment effect [11]. It has been used as an open-lung approach, especially in patients with early-stage ARDS, and has been reported to have various efficacies that reduce the number of ventilator management days and intensive care unit (ICU) stays and contribute to a reduction in sedative medications [4–7, 12]. However, universal indications of APRV other than ARDS are unclear, and previous reports that APRV can be a therapeutic ventilator setting in morbidly obese patients are limited [8–10].

In general, morbidly obese patients tend to have decreased chest wall compliance and respiratory muscle endurance. This occurs owing to anterior chest wall loading, which increases respiratory work and airway resistance and decreases basal lung capacity owing to visceral adipose tissue accumulating in the abdominal wall and cavity. This accumulation prevents diaphragmatic movement, obstructs peripheral airways, and causes a ventilation-perfusion (V/Q) mismatch [1, 2]. The patient was morbidly obese with diaphragmatic nerve palsy, causing the left diaphragm to move toward the thoracic cavity more easily. Furthermore, we thought that pneumonia and atelectasis resulted in respiratory failure owing to an inability to compensate for systemic oxygen demands.

The patient’s improved oxygenation could be attributed to APRV, with the initial intensive care interventions, such as multiple bronchoscopies, aggressive repositioning, and antibiotic therapy, providing foundational support. These interventions effectively addressed alveolar hypoventilation, corrected the V/Q mismatch, and resolved atelectasis.

In addition, clinical findings did not improve even though PCV mode was set to relatively high inspiratory pressure and PEEP, probably suggesting that continuous high positive pressure, such as APRV, might be effective in morbidly obese patients.

Furthermore, chest X-ray findings on the second and seventh days showed improvement in left lung atelectasis; however, oxygenation did not improve (Fig. 2). Although this could have been related to worsening atelectasis in the right lung, we considered the possibility of a phenomenon of similar to hypoxic pulmonary vasoconstriction occurring in the left lung [13]. Kozian *et al*. reported in an animal study examining V/Q mismatch after one-lung ventilation, in which, after 90 minutes of one-lung ventilation, the tidal volume returned to baseline values resuming both lung ventilation; on the other hand, the blood perfusion in the collapsed lung did not return to the initial baseline value after 90 minutes from restarting both lung ventilation [14]. Although it was unclear how long the nonaerated left lung continued, we postulated that the V/Q mismatch persisting after improvement in atelectasis may have prevented improvement in oxygenation. A previous report indicated APRV contributes secondarily to the redistribution of blood perfusion in the recruited area [11]; it could have contributed to correcting the continued V/Q mismatch in the left lung.

Throughout the treatment course, a series of interventions were methodically applied, each contributing incrementally to the patient’s overall respiratory improvement. These interventions included bronchoscopy, active repositioning after tracheostomy, and antibiotic therapy. Each of these steps was vital in stabilizing the patient and improving lung function.

However, a significant observable change in the patient’s oxygenation was noted after the switch from PCV to APRV, as evidenced by the sharp increase in the P/F ratio depicted in Fig. 2. While it was crucial to recognize that the cumulative effect of all interventions likely facilitated this improvement, the extent of the change suggested a substantial role for APRV. This marked improvement indicates that APRV might be particularly effective in this morbidly obese patient.

Since we expected resolving atelectasis and pneumonia to improve oxygenation initially, we implemented other interventions, including bronchoscopy, tracheostomy, and antibiotic therapy. These approaches eventually led to delays in applying APRV, so the duration of ventilator management and length of hospital stay could have been reduced even more by the earlier switch to APRV mode.

Other approaches, such as prone position and high PEEP to improve oxygenation [15], would be considered. However, we thought prone positioning, a well-documented strategy for improving V/Q mismatch, might be associated with risks related to pressure sores, high intra-abdominal pressure and vascular access and bloodstream infection [15, 16], so we postponed this method initially. We employed lung recruitment maneuvers and adjusted PEEP levels cautiously, balancing the need for alveolar recruitment with the risk of barotrauma and hemodynamic instability, particularly in a patient with high intra-abdominal pressure owing to obesity. The initial conservative PEEP settings were part of our strategy to avoid potential complications while assessing the patient’s response to standard ventilatory settings.

The decision to eventually implement APRV could be a relatively secure approach to respiratory failure owing to the morbidly obese patient. This mode seemed to contribute to achieving both recruitment and appropriate oxygenation without the risks associated with high PEEP settings and without the complexities of managing prone positioning in a morbidly obese patient.

After the switch to APRV mode, the patient was managed in a sitting position with light sedation, and active respiratory rehabilitation was possible. Furthermore, other multidisciplinary treatments, including weight loss by nutritional management, were considered to have prevented the recurrence of respiratory failure even after APRV mode was ended. In this sense, APRV should be viewed as a therapeutic approach for patients with morbidly obesity-related respiratory failure from an early stage in critical care.

## Conclusion

We experienced a case in which oxygenation was markedly improved after ventilator management with APRV in a patient with morbid obesity and left diaphragmatic nerve palsy. While a multidisciplinary approach is essential for managing severe respiratory conditions in morbidly obese patients, APRV management may contribute directly to improving oxygenation in morbid obesity with respiratory failure and should be recognized as one of the therapeutic measures from the early stage of critical care.

## Data Availability

Not applicable.

## Abbreviations

APRV: Airway pressure release ventilation
ARDS: Acute respiratory distress syndrome
BMI: Body mass index
P/F ratio: PaO_2_/F_I_O_2_ ratio
V/Q mismatch: Ventilation-perfusion mismatch
PCV: Pressure–control ventilation
